# Rational design of a deuterium-containing M2-S31N channel blocker UAWJ280 with *in vivo* antiviral efficacy against both oseltamivir sensitive and -resistant influenza A viruses

**DOI:** 10.1080/22221751.2021.1972769

**Published:** 2021-09-14

**Authors:** C. Joaquín Cáceres, Yanmei Hu, Stivalis Cárdenas-García, Xiangmeng Wu, Haozhou Tan, Silvia Carnaccini, L. Claire Gay, Ginger Geiger, Chunlong Ma, Qing-Yu Zhang, Daniela Rajao, Daniel R. Perez, Jun Wang

**Affiliations:** a Department of Population Health, College of Veterinary Medicine, University of Georgia, Athens, GA, USA; b Department of Pharmacology and Toxicology, College of Pharmacy, The University of Arizona, Tucson, AZ, USA; c Tifton diagnostic laboratory, College of Veterinary Medicine, University of Georgia, Tifton, GA, USA

**Keywords:** Influenza, M2 channel, antiviral, amantadine, drug resistance

## Abstract

Seasonal influenza A virus (IAV) infections are among the most important global health problems. FDA-approved antiviral therapies against IAV include neuraminidase inhibitors, M2 inhibitors, and polymerase inhibitor baloxavir. Resistance against adamantanes (amantadine and rimantadine) is widespread as virtually all IAV strains currently circulating in the human population are resistant to adamantanes through the acquisition of the S31N mutation. The neuraminidase inhibitor-resistant strains also contain the M2-S31N mutant, suggesting M2-S31N is a high-profile antiviral drug target. Here we report the development of a novel deuterium-containing M2-S31N inhibitor UAWJ280. UAWJ280 had broad-spectrum antiviral activity against both oseltamivir sensitive and -resistant influenza A strains and had a synergistic antiviral effect in combination with oseltamivir in cell culture. *In vivo* pharmacokinetic (PK) studies demonstrated that UAWJ280 had favourable PK properties. The *in vivo* mouse model study showed that UAWJ280 was effective alone or in combination with oseltamivir in improving clinical signs and survival after lethal challenge with an oseltamivir sensitive IAV H1N1 strain. Furthermore, UAWJ280 was also able to ameliorate clinical signs and increase survival when mice were challenged with an oseltamivir-resistant IAV H1N1 strain. In conclusion, we show for the first time that the M2-S31N channel blocker UAWJ280 has *in vivo* antiviral efficacy in mice that are infected with either oseltamivir sensitive or -resistant IAVs, and it has a synergistic antiviral effect with oseltamivir.

## Introduction

Influenza A virus (IAV) infections cause between 3 and 5 million severe infections with 300,000–650,000 deaths every year [1]. Although vaccination is considered the first line of defence against IAV, viral antigenic drift leads to annual vaccine updates and re-vaccination. Since current vaccine efficacy is at <50% [2,3], complementary anti-viral strategies that reduce disease burden due to influenza are highly desirable. Current antivirals help with viral clearance, and reduce transmission and deaths associated with influenza (reviewed in [4]). Currently, FDA-approved antiviral treatments designed specifically against IAV include neuraminidase inhibitors (NAI, oseltamivir, zanamivir and peramivir), M2 inhibitors (amantadine and rimantadine), and more recently the cap-dependent endonuclease inhibitor targeting the PA polymerase subunit (baloxavir [5]). Of these types of antivirals, NAIs are the antivirals of choice against current IAV strains. However, the presence of NAI-resistant H1N1 strains, particularly arising during the treatment of immunocompromised patients, is a reason for concern [6–9]. On the other hand, adamantanes exert its antiviral activity by blocking the M2 ion channel activity, which is required for the acidification of the virus interior after endocytosis and to trigger the release of the viral ribonucleoprotein particles out of the endosome [10–13]. Unfortunately, nearly 99% of IAV strains currently circulating in humans are resistant to adamantanes due to the acquisition of mutations that prevent drug binding to M2 but still allow the ion channel to remain active [12]. Among those mutations, the serine to asparagine mutation at residue 31 (S31N) is the most predominant associated with resistance to adamantanes [14,15]. Due to the widespread circulation of adamantane-resistant strains, the CDC does not recommend the use of adamantanes in the human population [4,16]. More recently, novel M2-S31N inhibitors have been developed showing promising results *in vitro* [14,15,17–24]. Here we report on the first M2-S31N inhibitor UAWJ280 with *in vivo* antiviral efficacy in an IAV-infected mouse model study. UAWJ280 is a deuterium-containing compound designed to block the M2-S31N proton channel and has been shown to inhibit both oseltamivir sensitive and -resistant IAVs. Combination of UAWJ280 with oseltamivir showed a synergistic antiviral effect in cell culture. UAWJ280 is well tolerated in mice and has favourable *in vivo* pharmacokinetic (PK) properties. More importantly, UAWJ280 showed antiviral activity alone or in combination with oseltamivir *in vivo* in mice challenged with a lethal dose of a prototypic 2009 pandemic H1N1 IAV strain A/California/04/2009 (H1N1) (Ca/04), which carries the M2-S31N mutant and is naturally resistant to adamantanes. Furthermore, UAWJ280 provided significant protection against an oseltamivir-resistant H1N1 IAV strain A/California/04/2009 (H1N1)-H275Y (Ca/04 OsR), suggesting that UAWJ280 offers a complementary alternative when NAI inhibitors are ineffective. Overall, UAWJ280 represents the first M2-S31N inhibitor with *in vivo* antiviral efficacy against both oseltamivir-sensitive and -resistant IAVs.

## Materials and methods

**Ethics statement on animal use and compliance.** Animal studies were approved by the Institutional Animal Care and Use Committee (IACUC) at the University of Georgia (Protocol A2020 07-004-Y1-A0). Experiments were performed under animal biosafety level 2 conditions. Animal studies and procedures were performed according to the Institutional Animal Care and Use Committee Guidebook of the Office of Laboratory Animal Welfare and PHS policy on Humane Care and Use of Laboratory Animals. Animal studies were carried out in compliance with the ARRIVE guidelines (https://arriveguidelines.org). Five- to 6-week-old female BALB/c mice were purchased from Jackson Laboratories (Bar Harbor, ME). Mice that lost ≥25% of their initial body weight (a score of 3 on a 3-point scale of disease severity) were humanely euthanized. Animals were humanely euthanized following guidelines approved by the American Veterinary Medical Association (AVMA).

**Viruses**. Influenza A viruses A/Switzerland/9715293/2013 X-247 (H3N2), FR-1366, A/Washington/29/2009 (H1N1), FR-460, A/North Carolina/29/2009 (H1N1), FR-488, and A/California/07/2009 (H1N1), FR-201, were obtained through the Influenza Reagent Resource, Influenza Division, WHO Collaborating Center for Surveillance, Epidemiology and Control of Influenza, Centers for Disease Control and Prevention, Atlanta, GA, USA. Influenza virus A/Denmark/528/2009 (H1N1) was obtained from Dr Elena Govorkova at St. Jude Children’s Research Hospital. Influenza virus A/Texas/04/2009 (H1N1) was obtained from Dr James Noah at the Southern Research Institute. The following reagent was obtained through BEI Resources, NIAID, NIH: Influenza A Virus, A/Wisconsin/67/2005 (H3N2), NR-41800. The mouse-adapted pandemic-origin A/California/04/2009 (H1N1) (Ca/04) has been previously described [25]. To obtain the Ca/04 virus resistant to oseltamivir (Ca/04 OsR), site-directed mutagenesis was performed on the pDP NA Ca/04 reverse genetics plasmid to introduce the H275Y (843-cac-845 to 843-tac-845 codon) mutation. The resulting pDP NA H275Y plasmid was paired with the remaining seven reverse genetics plasmids encoding the rest of the Ca/04 genome and rescued by reverse genetics as previously described [25]. Virus stocks were amplified in 10-day-old specific-pathogen-free (SPF) embryonated chicken eggs and stored at −80°C until use. The genome sequence of the Ca/04 was verified by Illumina’s MiSeq next generation sequencing as described [26]. The NA H275Y mutation in the Ca/04 OsR virus was verified by the Sanger sequence (Psomagen, Rockville, MD).

**UAWJ280 synthesis and characterization.** UAWJ280 was synthesized using the reduction amination procedure reported earlier [17,23]. The purity and identity of this compound was characterized by HNMR, CNMR and mass spectrometry. 3-{[(5-iodothiophen-2-yl)(D)methyl]amino}adamantan-1-ol (UAWJ280). Yield: 78%. ^1^H NMR (500 MHz, CD3OD) δ 7.09 (d, *J* = 3.6 Hz, 1H), 6.69 (dd, *J* = 3.6, 0.9 Hz, 1H), 3.90 (s, 1H), 3.35 (s, 1H), 2.30–2.19 (m, 2H), 1.71–1.60 (m, 11H), 1.58–1.52 (m, 2H). ^13^C NMR (126 MHz, CD3OD) δ 151.85, 137.80, 127.99, 72.83, 70.01, 55.38, 50.18, 49.85, 45.05, 41.72, 36.28, 32.14. C15H19DINOS, [M + H^+^] calculated 391.3, found 391.0.

**Formulation of antiviral compounds.** UAWJ280 was dissolved to the desire concentration in a suspension of 10% DMSO/90% corn oil. Oseltamivir phosphate (Sigma, SML1606) was dissolved to the desire concentration in sterile distilled water. Oseltamivir carboxylate was synthesized by ester hydrolysis of oseltamivir phosphonate. Methanol and water (LC-MS grade) were purchased from Fisher Scientific (Fair Lawn, NJ). Formic acid, nifedipine and β-Nicotinamide adenine dinucleotide 2′-phosphate reduced tetrasodium salt hydrate (NADPH) were purchased from Sigma-Aldrich (St. Louis, MO).

**Two-Electrode Voltage Clamp (TEVC) Assay.***In vitro* channel blockage of A/California/07/09 (H1N1) M2 WT, L26I, L46P and L26I/L46P by UAWJ280 was tested in a two-electrode voltage clamp assay using *Xenopus laevis* frog oocytes microinjected with corresponding RNAs as previously reported [15,27,28]. In brief, L26I, L46P, and L26I/L46P mutants were generated via Quikchange site-directed mutagenesis according to the manufacturer protocol (Agilent Technology). The primers are available upon request. The potency of UAWJ280 against various Cali M2 variants was determined by measuring their K_d_ values. The detailed procedure was described in our previous publication [28]. Briefly, a washout protocol was applied at the end of the application of the compound. During the washout period, we applied a pH 5.5 pulse instead of continuous application of pH 5.5 barth solution in order to prevent prolonged acidification of oocytes. The inhibition and washout curves were fitted into the association then dissociation equation in GraphPad Prism 8 [18]. UAWJ280 against mutant M2 variants L26I, L46P, and L26I/L46P displayed quick on and quick off phenotypes and their IC_50_ values are deemed as K_d_ values.

**Animals and treatments for the *in vivo* PK studies.** Male BALB/c mice (20–22 g) were purchased from Jackson Laboratories (Bar Harbor, ME). All mice were housed under conditions of controlled temperature (22°C) with on–off light cycle, with food and water provided ad libitum. For a pharmacokinetic study of UAWJ280, male BALB/c mice were given the compounds at 50 mg/kg (in corn oil containing 10% DMSO) by intraperitoneal injection (i.p.) injection. All animal studies were approved by the University of Arizona Animal Care and Use Committee.

**Sample preparation for pharmacokinetic analysis.** Blood samples were collected through the tail vein using heparinized capillary tubes at various times as indicated in the figure legends after dosing. Plasma was prepared and stored at −30°C until analysis. For the analysis of UAWJ280, plasma samples (5 µL each) were mixed with 30 µL methanol and 10 µL of the IS (internal standard UAWJ102, 1 µM). The mixture was vortexed for 30 s, then was diluted with 760 µL Milli Q water and loaded onto an OASIS® HLB cartridge (C18, 1 ml/30 mg, Waters Corporation, Milford, MA) pre-conditioned with 1 mL methanol followed by 1 mL water and the cartridge was then washed with 1 mL water. The analytes were finally eluted from the cartridges with 1 ml methanol, dried with nitrogen, and reconstituted in 100 µL methanol for the LC–MS analysis. The pharmacokinetic parameters were calculated using a PK solver (Microsoft, Redmond, WA), by assuming a noncompartmental model.

**Evaluation of *in vivo* toxicity.** Mice were randomly distributed into different groups (*n* = 2/group). Administration of antiviral compounds was performed for seven days, twice daily through intraperitoneal injections (I.P). UAWJ280 at 200 mg/kg/day was administered in 10% DMSO/90% corn oil suspension, 50 µl/mouse at a dose of 100 mg/kg. Negative control mice received the vehicle alone (10% DMSO/90% corn oil suspension). Oseltamivir at 10 mg/kg/day was administered in a final volume of 50 µl/mouse at a dose of 5 mg/kg. Combination therapy consisted of UAWJ280 (100 mg/lg/dose) plus oseltamivir (5 mg/kg/dose) in a final volume of 100 µl/mouse (1:1 suspension prepared at the time of inoculation). On day 10 post-drug administration, mice were humanely euthanized. Brain, liver, kidney, heart and lungs were collected post-mortem from all mice for histopathological analyses.

**Evaluation of antiviral activity *in vivo*.** The mice were randomly distributed into the different groups, anaesthetized with isoflurane and subsequently inoculated via the intranasal route with 50 µl of an inoculum containing either PBS (mock group) or 5 mouse lethal dose 50 (MLD50, ∼5 × 10^3^ tissue culture infectious dose 50 (TCID50)/mouse) of either the wild-type Ca/04 or the Ca/04 OsR virus. After inoculation, the mice were monitored daily for clinical signs of disease, body weight loss, and mortality. Antiviral treatment was initiated at 4 h post-challenge (hpc) and maintained for 7 days, twice daily, through intraperitoneal injections (I.P) as described above. Three experiments were performed using the wild-type Ca/04 as the challenge virus. In experiment 1, the following groups of mice and treatment groups were tested (*n* = 6/group): vehicle alone (10% DMSO/90% corn oil suspension)-mock challenge, vehicle alone (10% DMSO/90% corn oil suspension)-Ca/04 challenge, UAWJ280 (200 mg/kg/day)-Ca/04 challenge, Oseltamivir (10 mg/kg/day)-Ca/04 challenge and Combination therapy (UAWJ280 at 100 mg/kg/dose plus oseltamivir at 5 mg/kg/dose; ratio 1:1)-Ca/04 challenge. In experiment 2, the vehicle alone (10% DMSO/90% corn oil suspension)-Ca/04 challenge and UAWJ280 (200 mg/kg/day)-Ca/04 challenge groups were evaluated (*n* = 6/group). For experiment 3 (*n* = 8/group), the same groups described for experiment 1 were evaluated and subsets of mice (*n* = 4/group) were humanely euthanized at 7 and 14dpc and lungs and nasal turbinates (NT) were collected. At 21 dpc, the surviving mice in experiments 1 and 2 were euthanized, and serum samples were collected to evaluate virus-specific antibody responses. One experiment was performed using the Ca/04 OsR virus in which the following groups were tested (*n* = 6/group): vehicle alone (10% DMSO/90% corn oil suspension)-mock challenge, vehicle alone (10% DMSO/90% corn oil suspension)-Ca/04 OsR challenge, UAWJ280 (200 mg/kg/day)-Ca/04 OsR challenge and Oseltamivir (10 mg/kg/day)-Ca/04 OsR challenge. At 21 dpc, the surviving mice were euthanized, and serum samples were collected to evaluate virus-specific antibody responses.

**HI assays.** Serum samples collected at 21 dpc were screened for the presence of neutralizing antibodies by the HI assay using the wild-type Ca/04 or the Ca/04 OsR strain. Briefly, the sera were treated with a receptor-destroying enzyme at 37°C overnight and then heat inactivated at 56°C for 30 min. Then, the sera were diluted 1:10 with PBS and subsequently serially diluted 2-fold and mixed with 4 hemagglutination units (HAU) of virus in a 96-well plate and incubated at room temperature for 15 min. The HI activity was visualized by adding 0.5% turkey red blood cells to the virus-serum mixtures and further incubation at room temperature for 30 min before reading.

***Titration samples.*** Tissue homogenates were generated using the Tissue Lyzer II (Qiagen). Briefly, 500 ul of PBS-AB was added to each sample together with Tungsten carbide 3 mm beads (Qiagen). Samples were homogenized during 15’ and centrifuged at 15,000 g for 10 min. Supernatants were collected, aliquoted and stored at −80°C until further analysis. Samples were titrated by tissue culture infectious dose 50 (TCID_50_) and virus titres were established by the Reed and Muench method [29].

***Histopathology.*** Selected tissues, including lungs, nasal turbinates (NT), kidney, liver, heart, and brain were collected from a representative number of mice in each group and at different timepoints for histopathological examination. Tissues were placed in 10% neutral-buffered formalin (NBF), fixed for at least 72 h, paraffin embedded and processed for routine histopathology with hematoxylin and eosin staining (HE). Tissues were subjectively scored by a pathologist blinded to the study as none (0), mild; ≤10% (1), mild to moderate; 11–25% (2), moderate; 26–40% (3), moderate to severe; 41-60% (4) and severe ≥60% (5) based on lesion severity and extent of inflammation. Features considered for the scoring were the following: presence and extent of necrosis, haemorrhage, oedema (interstitial and/or alveolar), fibrin/hyaline membranes, pneumocyte type 2 hypertrophy and hyperplasia, mesothelial hyperplasia, bronchitis/bronchiolitis, bronchopneumonia, pleuritis and vasculitis. For immunohistochemistry (IHC) against IAV, a polyclonal antibody (Meridian Life Science; dilution 1/1,500) was used. The staining was used to estimate the amount of viral antigens. Staining intensity and distribution were subjectively scored by a pathologist blinded to the study using a scale from none (0) to large/highest positivity (5).

***Plaque assay.*** MDCK cell line or MDCK cell line expressing ST6Gal I in plaque assays to detect the antiviral activity of UAWJ280 in multiple influenza A strains was carried out as previously described [18,21,30–33]. Briefly, a confluent monolayer of ST6Gal I MDCK cells was incubated with ∼100 pfu virus/well in DMEM with 0.5% BSA at 4°C for 1 h followed by at 37°C for 1 h. The inoculums were then removed, and the cells were washed with phosphate-buffered saline (PBS). The cells were incubated with DMEM containing 1.2% Avicel microcrystalline cellulose (FMC BioPolymer, Philadelphia, PA), NAT (2.0 μg/mL), and different concentrations of the testing compound. After 2 days of incubation at 37°C with 5% of CO_2_ in the cell culture incubator, the overlay was removed and the cell monolayers were fixed and stained with crystal violet dye solution (0.2% crystal violet, 20% methanol). EC_50_s were determined by curve fittings obtained using log (concentration of inhibitors) vs. percentage of plaque area determined in Image J with variable slopes in prism 8.

***Serial viral passage experiment***. Serial viral passage experiments were carried out in the presence of increasing concentrations of UAWJ280, starting with a concentration of ∼1 × EC_50_ at P1. MDCK cells were infected with A/California/07/2009 (H1N1) at an MOI of 0.01, and the amplified virus in the cell culture supernatant was collected after approximately 48 h when a significant cytopathic effect was observed, and viral titer was quantified by plaque assay. Drug sensitivity was tested in the P6 and P12 by determining the EC_50_ values using plaque assay. The viral genome RNA was purified using the QIAGEN viral RNA mini kit, followed by reverse transcription using SuperScript III first-strand reverse transcriptase (Invitrogen) with primer (5′-AGCAAAAGCAGG-3′). The viral M fragment was amplified using specific primers (5′-TAGATATTAAAGATGAGTCTTC-3′ and 5′-CTCTAGCTCTATGTTGACAAAATGACC-3′) and the purified PCR product was sequenced by Eton Biosciences, Inc.

***Combination therapy***. The combinational antiviral effects of UAWJ280 and oseltamivir were evaluated in cell cultures as described previously [34,35] using plaque assay. UAWJ280 was mixed with oseltamivir at EC_50_ ratios of 8:1, 4:1, 2:1, 1:1, 1:2, 1:4, and 1:8, separately. Seven 3-fold serial dilutions of the mixture of UAWJ280 and oseltamivir at each combination ratio were applied in plaque assay to determine EC_50_ values of UAWJ280 and oseltamivir. The combination index (CI) plot was performed in Prism 8 to determine the EC_50_ values of UAWJ280 and oseltamivir at different combination ratios. The fractional inhibitory concentration index (FICI) was calculated using the following formula: FICI = (EC_50_ of UAWJ280 in combination)/(EC_50_ of UAWJ280 alone)+(EC_50_ of oseltamivir in combination)/(EC_50_ of oseltamivir alone). FICI<0.5 was interpreted as a significant synergistic antiviral effect [36].

**Graphs/Statistical analyses.** All data analyses and graphs were performed using GraphPad Prism software version 8 (GraphPad Software Inc., San Diego, CA). For multiple comparisons, a two-way analysis of variance (ANOVA) was performed. A *P-*value below .05 was considered significant.

## Results

### Design, synthesis, and *in vitro* antiviral activity of UAWJ280

**Figure 1.**
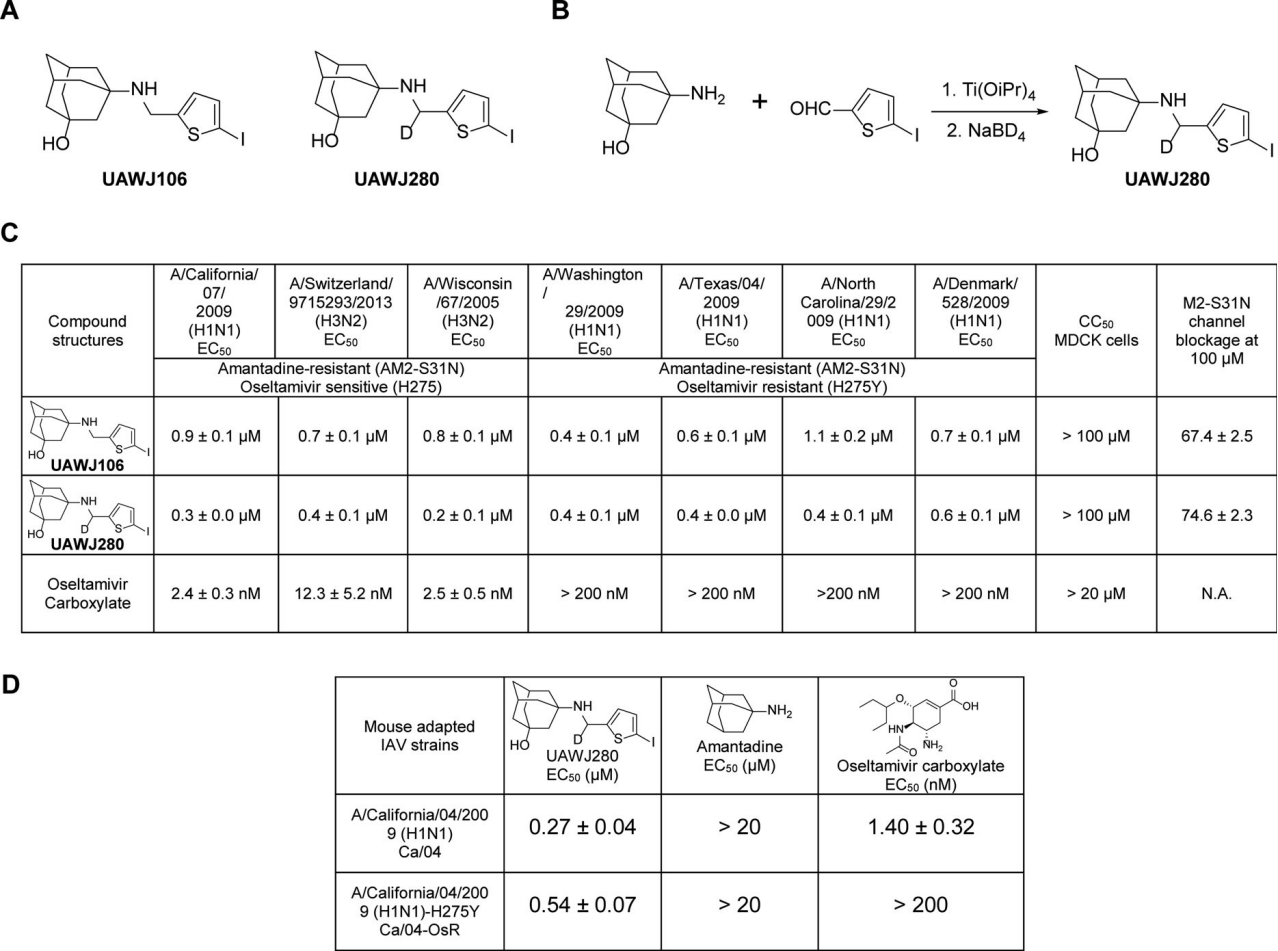
Synthesis and antiviral activity of UAWJ280. (A) Structures of UAWJ106 and UAWJ280. (B) Synthesis of UAWJ280. (C) Antiviral activity of UAWJ280 against clinical isolates of IAVs. ^a^Results for UAWJ106 and oseltamivir carboxylate were obtained from the previous study [17]. (D) Antiviral activity of UAWJ280 against mouse-adapted IAVs used in the *in vivo* study.

UAWJ280 was designed based on UAWJ106 (compound 10e in the original publication), which has favourable *in vitro* PK properties (Figure 1(A)) [17]. A deuterium was incorporated at the methylene linker with an aim to further improve the PK properties. UAWJ280 was synthesized by the optimized reduction amination procedure as reported before (Figure 1(B)) [24]. Two-electrode voltage clamp assay showed that UAWJ280 had improved channel blockage against the wild-type A/California/04/2009 M2 channel (Cali M2 WT) than the non-deuterated analogue UAWJ106 (74.6% versus 67.4% at 100 µM) (Figure 1(C)). The antiviral activity of UAWJ280 was tested against several contemporary influenza A viruses, among which A/California/07/2009 (H1N1), A/Switzerland/9715293/2013 (H3N2), A/Wisconsin/67/2005 (H3N2) are amantadine resistant (M2-S31N) and oseltamivir sensitive (H275); and A/Washington/29/2009 (H1N1), A/Texas/04/2009 (H1N1), A/North Carolina/29/2009 (H1N1), and A/Denmark/528/2009 (H1N1) are amantadine resistant (M2-S31N) and oseltamivir resistant (H275Y). It was found that UAWJ280 had broad-range antiviral activity against all IAVs tested with EC_50_ values ranging from 0.2 to 0.6 µM, and UAWJ280 was more potent than UAWJ106, which is consistent with its higher channel blockage than UAWJ106. The antiviral activity of UAWJ280 against the mouse-adapted IAV strains, the A/California/04/2009 (H1N1) (Ca/04) and the oseltamivir-resistant A/California/04/2009 (H1N1) (Ca/04 OsR), were also confirmed using the plaque assay, and the results were consistent with that from clinical isolates of IAVs (Figure 1(D)).

**Figure 2.**
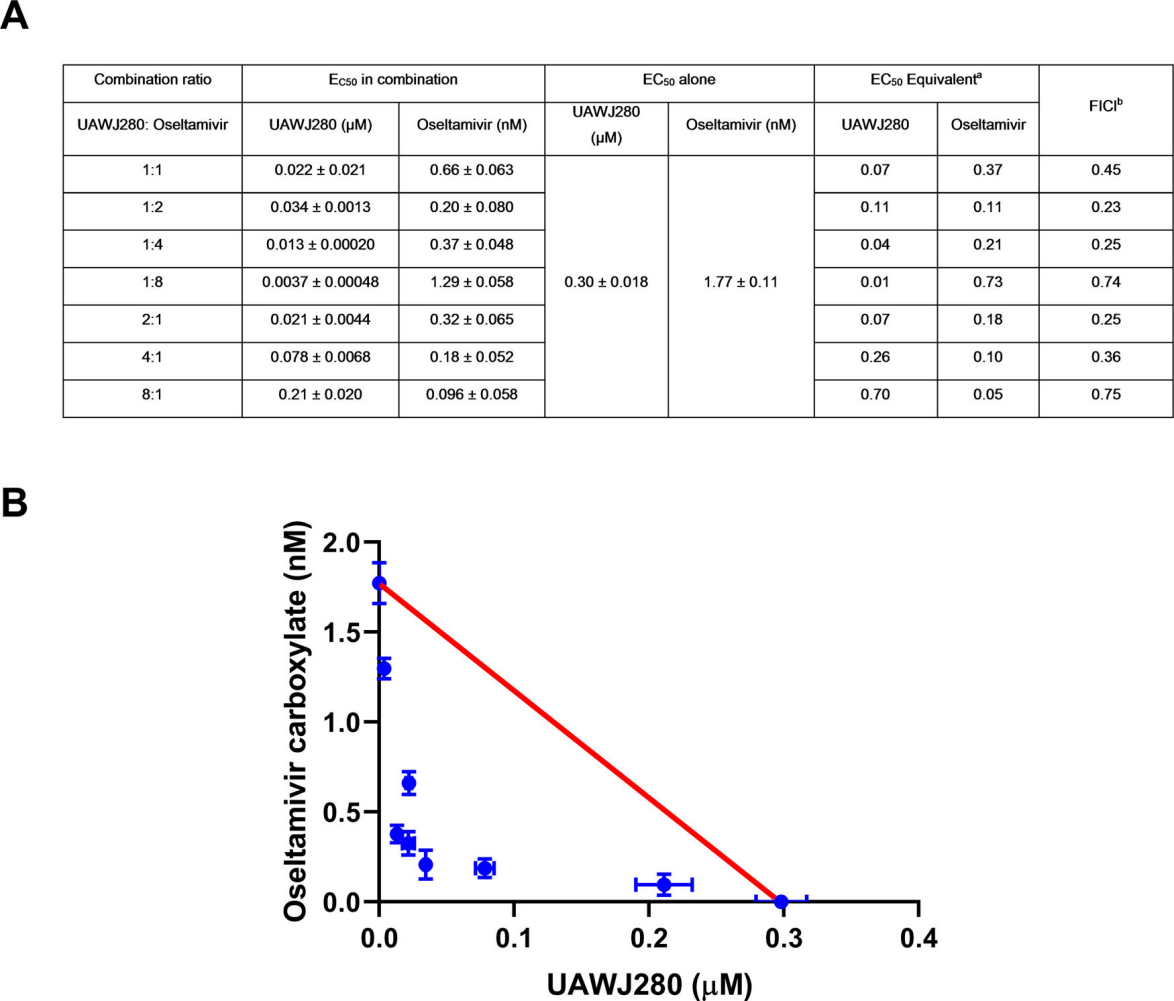
Combination therapy experiment of UAWJ280 with oseltamivir carboxylate. (A) Table of combination therapy with EC_50_ and FICI values. ^a^EC_50_ equivalent was the ratio of EC_50_ of the compound in each combination to its EC_50_ alone. ^b^FICI was the sum of UAWJ280 and oseltamivir EC_50_ equivalent in each combination. (B) Plot of combination indices (CIs) versus the EC_50_ values of the compounds at different combination ratios. Data are mean ± SD of two independent experiments.

As suggested by the FDA’s guidance for industry antiviral product development, combination therapy studies need to be performed for drug candidates in development with the current standard of care, which is oseltamivir in the case of influenza virus infection. As such, we performed the combination therapy experiment of UAWJ280 with oseltamivir carboxylate against the A/California/04/2009 (H1N1) virus in plaque assay using the fractional inhibitory concentration index (FICI) method (Figure 2(A)) [35]. In this experiment, UAWJ280 with oseltamivir carboxylate were mixed at different ratios and the corresponding EC_50_ and FICI values were calculated and plotted (Figure 2(A)). FICI values less than, equal to, or greater than 1 indicate synergy, additivity, or antagonism, respectively. Accordingly, the red line in Figure 2(B) indicates the additive effect; the upper right area above the red line indicates antagonism, and the bottom left area below the red line indicates synergy. It was found that the combination indices at all the combination ratios tested fell below the red line, suggesting UAWJ280 had a strong synergistic antiviral effect with oseltamivir.

### Profile the genetic barrier to drug resistance of M2-S31N channel blocker UAWJ280

**Figure 3.**
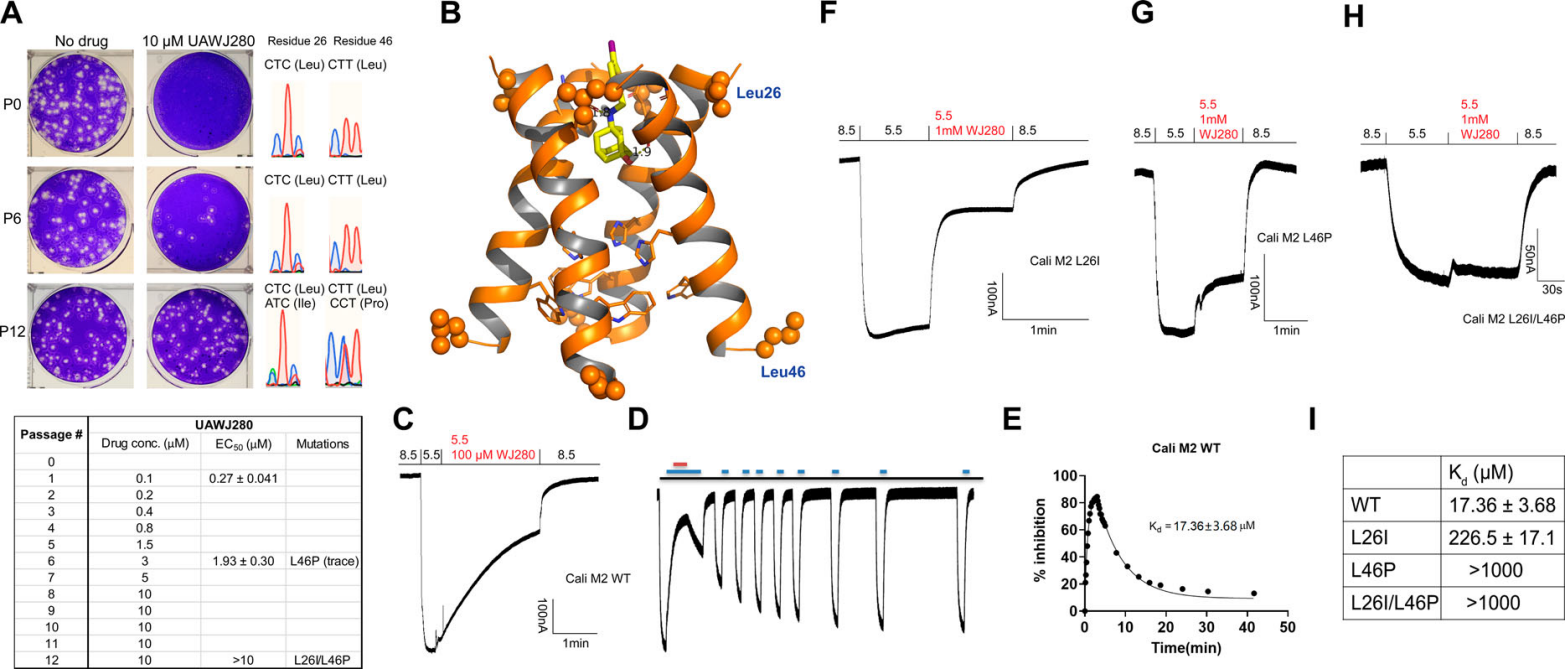
Profile the *in vitro* genetic barrier to drug resistance of UAWJ280. (A) Serial viral passage experiment to select drug-resistant mutants of A/California/07/2009 (H1N1) under the drug selection pressure of UAWJ280. The table shows the drug concentration applied in each passage. (B) Docking model of UAWJ280 in the M2-S31N channel. The docking pose was generated using Schrödinger Glide standard precision docking. Residues 26 and 46 were shown in spheres. Docking was performed using the M2-S31N structure with the PDB ID of 2LY0. (C) Electrophysiology recording trace of UAWJ280 in inhibiting Cali M2 WT. (D) Washout curve of UAWJ280 in inhibiting Cali M2 WT. Blue bar represents pH = 5.5 bath solution and red bar represents the addition of 100 μM of UAWJ280 in pH 5.5 bath solution. (E) K_d_ curve fitting of UAWJ280 in inhibiting Cali M2 WT using both the binding and washing curves. (F) Electrophysiology recording trace of UAWJ280 in inhibiting Cali M2 L26I. (G) Electrophysiology recording trace of UAWJ280 in inhibiting Cali M2 L46P. (H) Electrophysiology recording trace of UAWJ280 in inhibiting Cali M2 L26I/L46P. (I) K_d_ values of UAWJ280 in inhibiting Cali M2 WT and its mutants.

To independently characterize the antiviral mechanism of action of UAWJ280, we performed a serial viral passage experiment with the A/California/04/2009 (H1N1) virus under the escalating drug selection pressure of UAWJ280. It was found that the EC_50_ increased by 7-fold at passage 6 (1.93 µM at P6 vs. 0.27 µM at P0), and complete resistance was observed at passage 12 (EC_50_ > 10 µM) (Figure 3(A)). Sequencing the P6 and P12 genome revealed two M2 mutations, the L26I, and the L46P (Figure 3(A)). The L26I locates at the drug-binding site, while L46P locates at the allosteric site at the C-terminus of the channel (Figure 3(B)). To confirm the drug resistance, we generated three M2 mutants for the electrophysiology assay, the M2-L26I and M2-L46P single mutants, and the M2-L26I/L46P double mutants (Figure 3(C–I)). The K_d_ of UAWJ280 against Cali M2 WT is 17.36 ± 3.68 µM (Figure 3(C–E)). In comparison, the K_d_ values of UAWJ280 against the Cali M2-L26I, L46P, and L26I/L46P are 226.5 ± 17.1 µM, >1 mM, and >1 mM, respectively (Figure 3(F–I)). These results suggest that M2 is the drug target of UAWJ280, and both M2-L26I and L46P mutants confer drug resistance.

### *In vivo* pharmacokinetic (PK) profiling of UAWJ280

**Figure 4.**
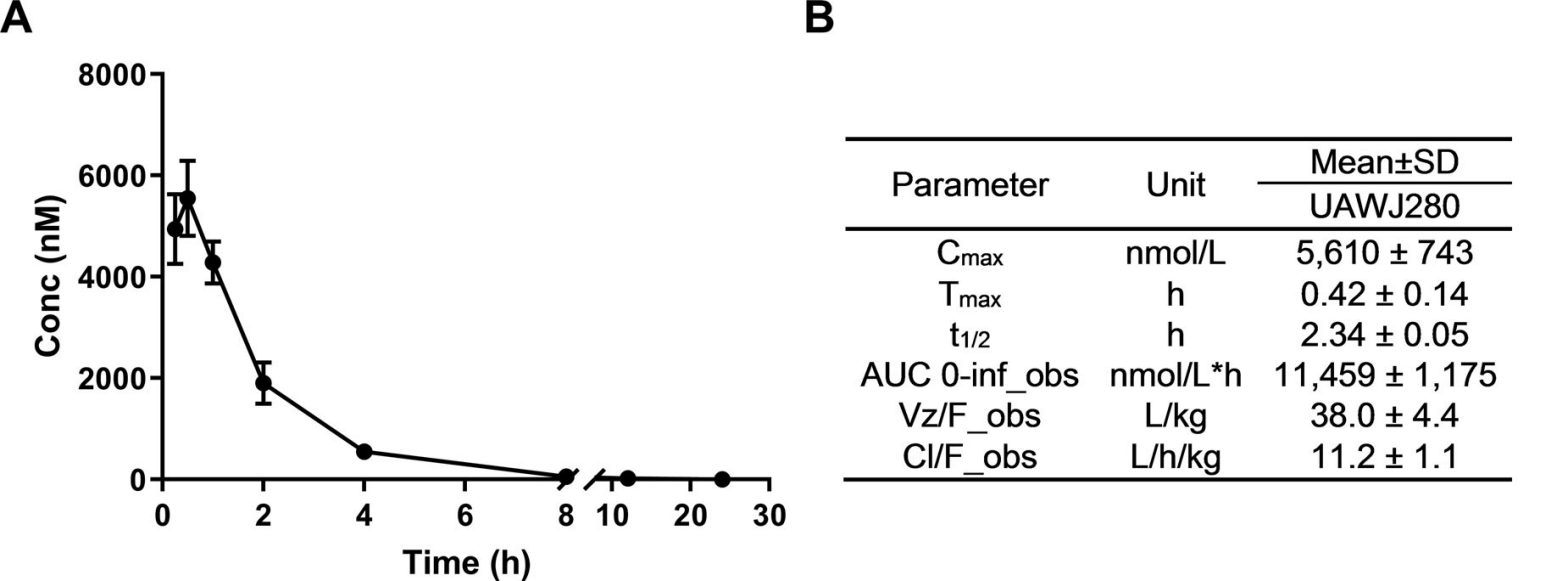
*In vivo* PK of UAWJ280 in BALB/c mice. (A) *In vivo* clearance of UAWJ280 in male BALB/c mice. (B) Pharmacokinetic parameters of UAWJ280 after I.P. injection at 50 mg/kg. The values represent means ± S.D., *n* = 3. Adult male BALB/c mice were given a single i.p. injection of UAWJ280 at 50 mg/kg. Blood was obtained at 0, 0.25, 0.5, 1, 2, 4, 8, 12 and 24 h after administration of the compound. The plasma concentration of UAWJ280 and the internal standard were analyzed by LC-MS/MS.

UAWJ280 was chosen for the *in vivo* PK studies as it had improved antiviral activity than UAWJ106. UAWJ280 was formulated in a 10% DMSO/90% corn oil suspension and was administrated to male BALB/c mice by intraperitoneal (i.p.) injection at 50 mg/kg. The plasma concentration of UAWJ280 was analyzed by LC-MS/MS using Multiple Reaction Monitoring (MRM) at *m/z* 390.9/223.8. For quantification of UAWJ280, a calibration curve was constructed using the pure UAWJ280 via linear regression. The correlation coefficient value UAWJ280 in plasma were >0.99 for a range of 10–4000 nM. The limit of quantification (LOQ) was 10 nM with signal to noise ratio >10:1. The recovery was more than 80% under the current sample preparation method. The pharmacokinetic profiles of UAWJ280 are shown in Figure 4. UAWJ280 had a rapid absorption after i.p. administration and reached the maximum plasma concentration at 0.42 h (Tmax), with a peak plasma concentration (Cmax) of 5610 nM. The clearance of UAWJ280 is also relatively fast, with a half-life (t_1/2_) of 2.34 h. The area under concentration time curve (AUC), the apparent volume of distribution (Vz/F) and the apparent clearance (CL/F) are 11,459 ± 1175 nmol/L*h, 38.0 ± 4.4 L/kg and 11.2 ± 1.1 L/h/kg, respectively.

### UAWJ280 does not cause signs of toxicity in mice

**Figure 5.**
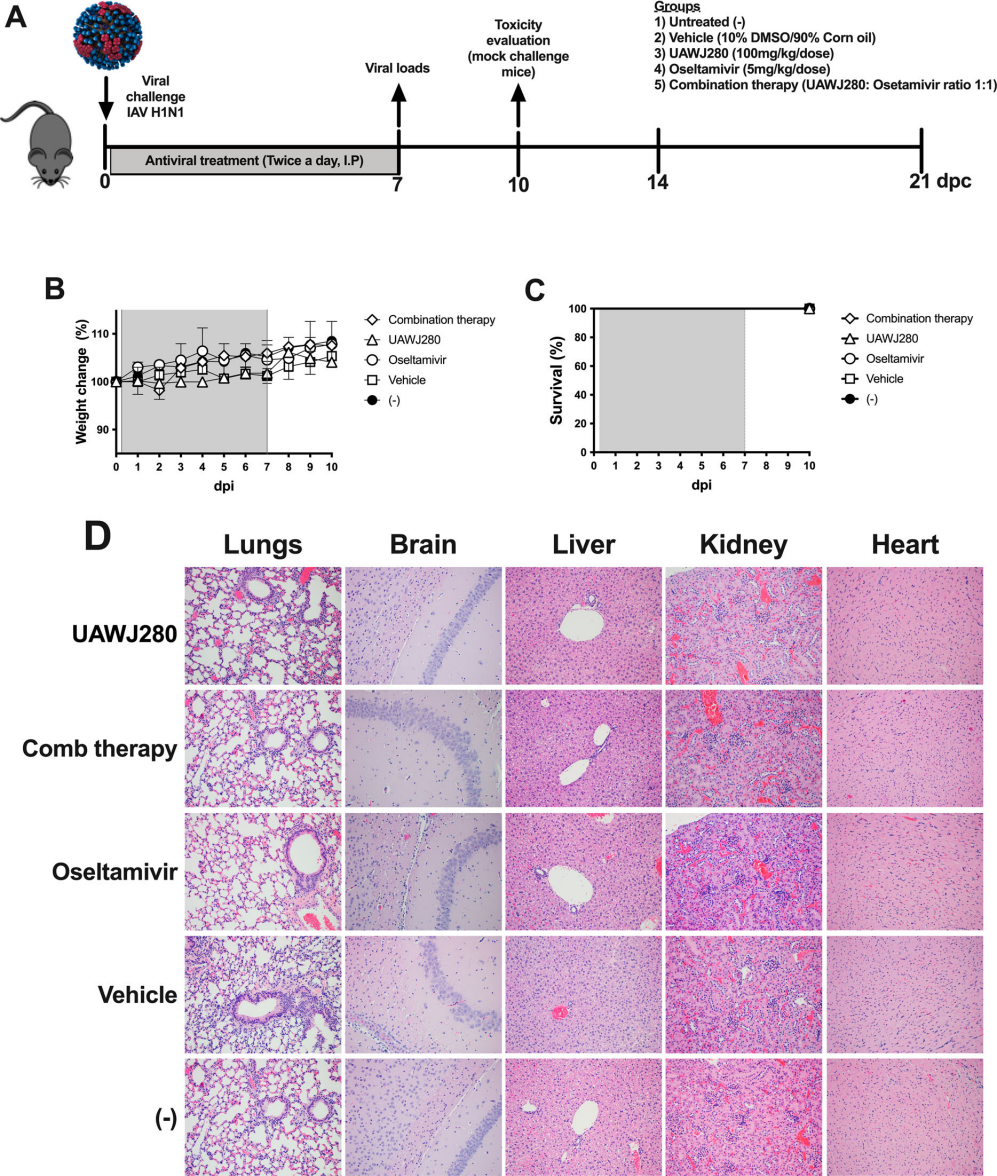
Evaluation of the safety profile of UAWJ280 alone or in combination with oseltamivir in mice model. Mice were non-treated (-), treated with drug vehicle, UAWJ280 (200 mg/kg/day), oseltamivir (5 mg/kg/day) and combination therapy (ratio 1:1 UAWJ280:oseltamivir) for 7 days, twice a day through intraperitoneal injection. (A) Schematic representation of the experiments. (B) Weight changes and (C) survival were monitored daily for 10 days. (D) Lungs, brain, liver, kidney and heart were obtained after 10 days of treatment and histopathological examination was performed to evaluate the presence of microscopical lesions. Representative pictures were taken at 20X.

The safety of UAWJ280 (200 mg/kg/day) *in vivo* in a mouse model was evaluated either alone or in combination with oseltamivir (10 mg/kg/day) and compared to the control group that received oseltamivir alone (Figure 5(A)). Additional controls included vehicle-treated (10% DMSO/90% corn oil suspension) and untreated mice. Mice were treated I.P. twice daily for seven days. Daily monitoring and clinical evaluations showed no differences in terms of clinical signs and weight changes among mice in the different antiviral regimes compared to the vehicle-treated or untreated negative control mice (Figure 5(B)). Survival of 100% was observed in all the groups (Figure 5(C)). Tissue samples collected on day 10 (3 days post final treatment) from brain, livers, kidneys, lungs and hearts showed no signs of toxicity associated with either UAWJ280, UAWJ280 plus oseltamivir or oseltamivir in comparison to the vehicle-treated or negative control groups. (Figure 5(D)). Taken together, these results suggest a lack of obvious toxicity in mice associated with treatment with UAWJ280 alone or in combination with oseltamivir.

### UAWJ280 shows antiviral activity *in vivo* against the oseltamivir sensitive Ca/04 (H1N1) virus in mice

**Figure 6.**
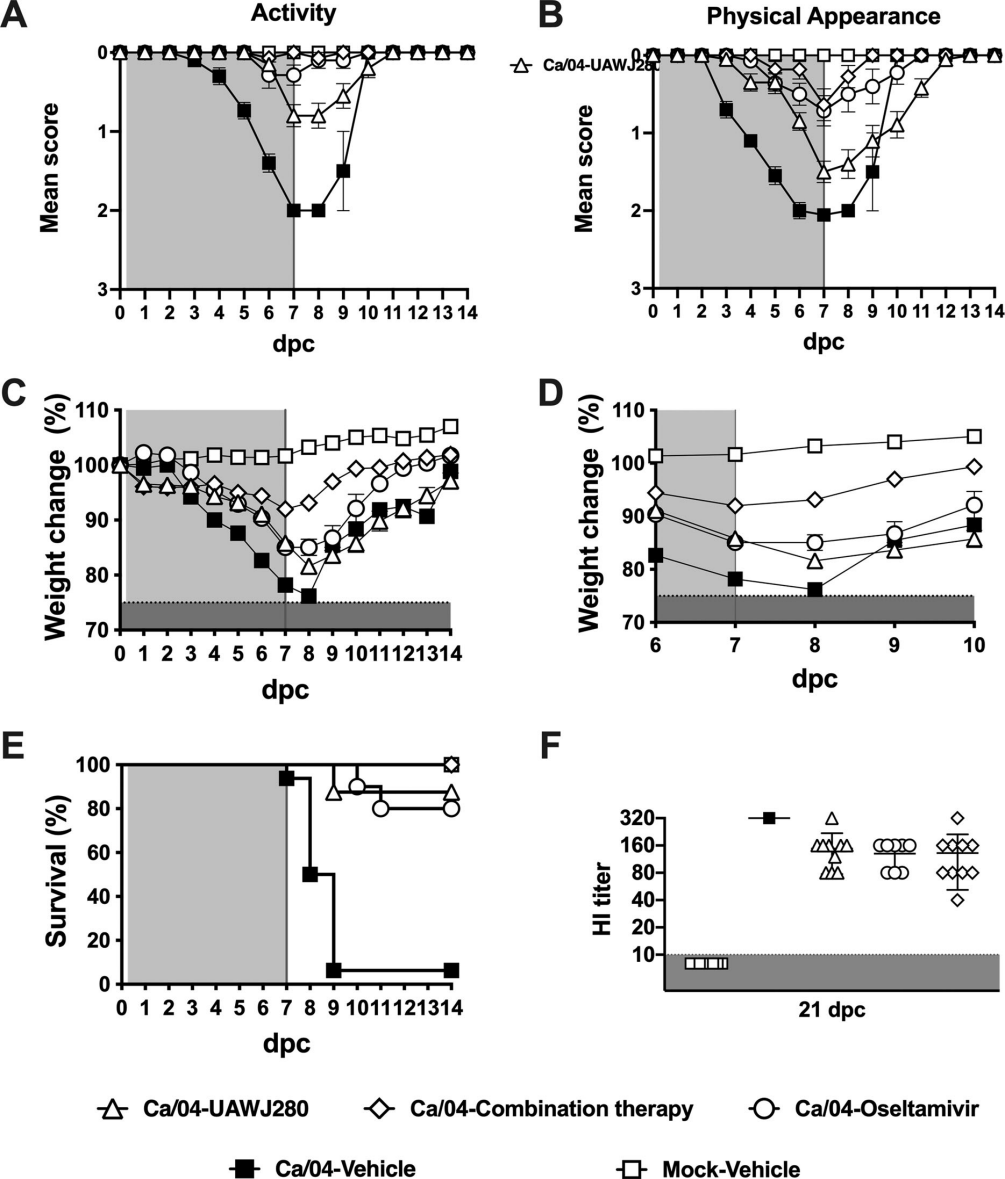
Evaluation of the efficacy of UAWJ280 alone or in combination with oseltamivir in the mice model. Mice were inoculated with 5xMLD50 of Ca/04 (H1N1) and treated with drug vehicle, UAWJ280 (200 mg/kg/day), oseltamivir (10 mg/kg) and combination therapy (ratio 1:1 UAWJ280:oseltamivir) through intraperitoneal injection 4 h post-challenge and continue for 7 days, twice a day (cyan shedding). Clinical signs related to (A) activity, (B) physical appearance, (C-D) weight change and (E) survival were evaluated among the different groups. (F) Sera were recovered at 21 dpc and the levels of neutralizing antibodies in each group were assessed using HI assay.

Mice were challenged with 5xMLD50 of the Ca/04 (H1N1) virus (Figure 6). It is important to note that the Ca/04 (H1N1) naturally contains the M2-S31N mutant that confers resistance to amantadine. Three independent experiments were performed. In the three experiments, antiviral treatment was initiated at 4 h post (virus) challenge and continued twice daily until day 7 post-challenge (dpc). Mice were monitored for clinical signs, body weight changes, and survival until 14 dpc (7 days post-treatment). The data presented below combines the analysis of the three experiments. The results of the three studies combined revealed that mice treated with UAWJ280 (*n* = 20) showed minor changes in activity but still responding well to changes in the environment. Mice in the combination therapy (*n* = 14) and oseltamivir-treated (*n* = 14) groups remained alert throughout the study and did not show any effects on activity compared to mock challenge-vehicle-treated controls (*n* = 14, Figure 6(A)). In contrast, mice challenged with Ca/04 (H1N1) but without antiviral treatment (*n* = 20) were the least active (highest score) and responded only after physical stimulation between 4 and 10 dpc. The physical appearance of mice in the combination therapy group was normal and indistinguishable from those in the mock-vehicle group. Mice treated with either UAWJ280 or oseltamivir showed mild clinical signs, although those in the UAWJ280 were more noticeable. However, mice that received antiviral treatment showed overall improved physical appearance and delayed clinical signs compared to the Ca/04-challenge, vehicle-treated mice. Ca/04-challenged mice without antiviral treatment showed lack of grooming and rough coat starting at 3 dpc (Figure 6(B)). Consistent with the observations of activity and physical appearance, mice that received the combination therapy showed <10% loss of the initial weight and 100% survival (Figure 6(C,D)), whereas those treated with either UAWJ280 or oseltamivir alone showed a similar decline in body weight (∼15%) and similar survival rates, 90% and 85%, respectively. In contrast, control mice in the Ca/04-challenge, vehicle-treated mice group showed the sharpest decrease in body weight (≥20% by 7 dpc) and survival below 10% (Figure 6(E)). To rule out the possibility that surviving mice were not properly challenged with the virus, sera were collected from all surviving mice at 21 dpc and used to detect the levels of neutralizing antibodies through HI assays. The results of the HI assays showed that all surviving mice had seroconverted, which is consistent with the notion that they were indeed infected and recovered from the Ca/04 virus challenge (Figure 6(F)) Taken together, these results demonstrate the protective effect of UAWJ280 against H1N1 IAV. A synergistic effect was observed when UAWJ280 was combined with oseltamivir treatment.

### UAWJ280 in combination with oseltamivir decreases Ca/04 (H1N1) viral load in mice

**Figure 7.**
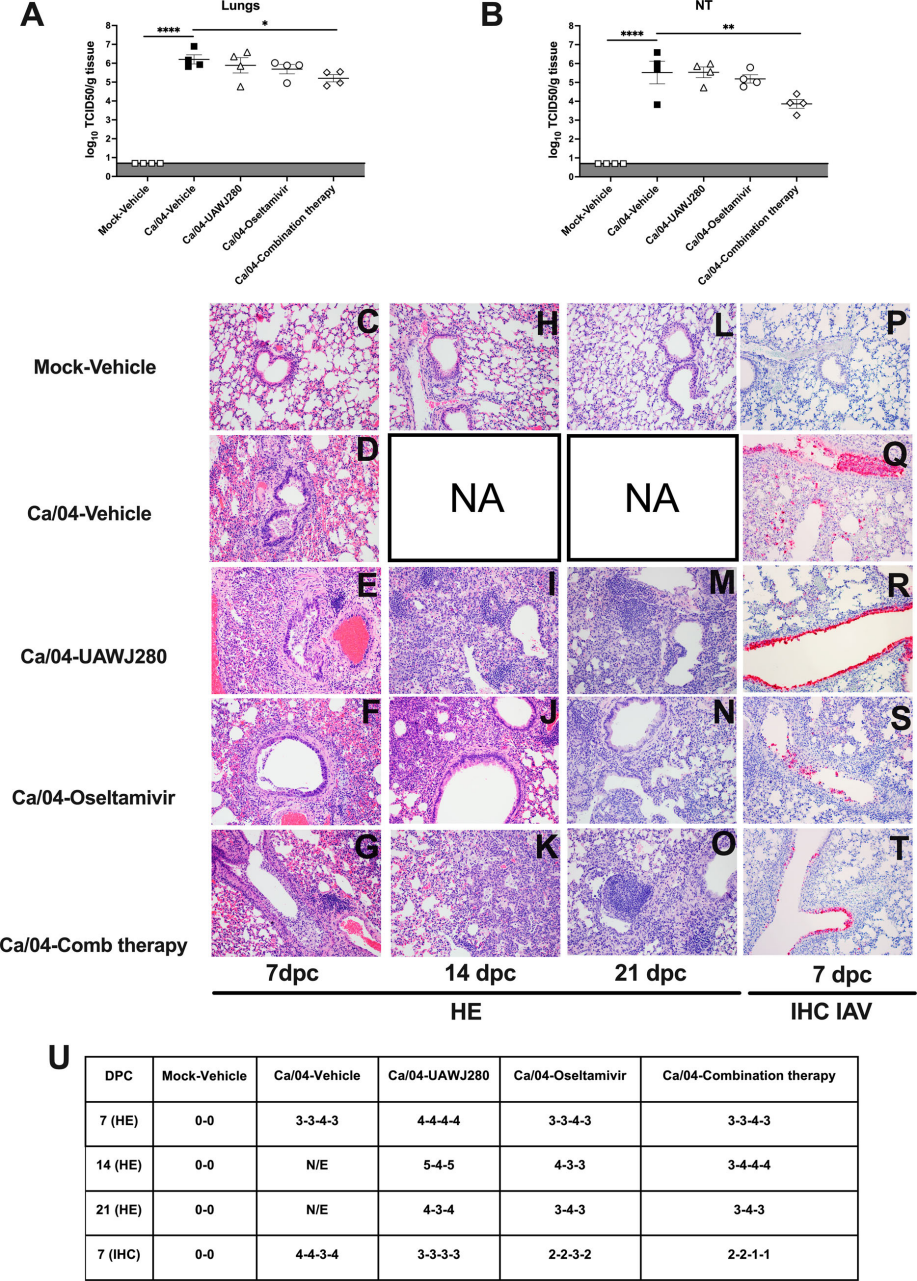
Viral loads, HE and IHC of mice treated with UAWJ280 alone or in combination with oseltamivir in the mice model. Mice were inoculated with 5xMLD50 of Ca/04 and treated with drug vehicle, UAWJ280 (100 mg/kg per dose), oseltamivir (5 mg/kg per dose) and combination therapy (ratio 1:1 UAWJ280:oseltamivir) through intraperitoneal injection 4 h post-challenge and continue for 7 days, twice a day. At 7 dpc, mice were euthanized, and tissues homogenates were prepared from lung and NT to evaluate the viral loads in (A) lungs and (B) NT. (C-O) Histopathology of lungs collected at 7 dpc (C-G), 14 dpc (H-K) and 21dpc (L-O). C. Mock-Vehicle at 7 dpc. Lung, normal; (D-G). Ca/04-Vehicle, Ca/04-UAWJ280, Ca/04-Oseltamivir and Ca/04-Comb therapy at 7dpc. Variable degree of subacute necrotizing bronchiolitis with intraluminal fibrinonecrotic exudate formation. Surrounding alveoli are infiltrated by mixed inflammatory cells, mostly neutrophils, macrophages and lesser lymphocytes and plasma cells. (H). Mock-Vehicle at 14 dpc. Lung, normal. (I). Ca/04-UAWJ280 at 14 dpc. Severe diffuse epithelization of the alveoli due to pneumocyte type 2 hyperplasia. Marked alveolar oedema, neutrophilic alveolitis and lymphoplasmacytic perivascular cuffs are observed throughout the parenchyma; (J-K). Ca/04-Oseltamivir and Ca/04-Comb therapy at 14 dpc. Moderate, multifocal, well-demarcated areas of consolidation due to pneumocyte 2 hyperplasia and lymphoplasmacytic and histiocytic inflammation are effacing the hilar areas. (L). Mock-vehicle at 21 dpc. Lung, normal; (M). Ca/04-UAWJ280 at 21 dpc. Alveolar septa are expanded by moderate to severe pneumocyte 2 hyperplasia. Moderate perivascular lymphoplasmacytic inflammation with occasional lymphoid nodule formation is present throughout the section. The bronchiolar epithelium is regenerating and occasionally blebbing and sloughing into the lumen. (N-O). Ca/04-Oseltamivir and Ca/04-Comb therapy at 21 dpc. Pneumocyte 2 hyperplasia and lymphoplasmacytic and histiocytic inflammation are moderate, well-demarcated and effacing hilar parenchyma. (P-T) IHC against Influenza A at 7dpc in lungs. (P) Mock-Vehicle. Lung, normal. (Q) Ca/04-Vehicle. Lung, moderate-severe virus nucleoprotein positivity (red) widespread distribution within the bronchiolar necrotic exudate, bronchiolar epithelial cells, and adjacent alveolar septa. (R) Ca/04-UAWJ280 Lung, bright diffuse moderate positivity (red) within selected bronchiolar epithelium. Small numbers of positive cells present within alveolar septa; (S) Ca/04-Oseltamivir and (T) Ca/04-Comb therapy. Lung, mild-moderate positivity mostly confined to epithelial cells of bronchioles. (U) HE and IHC scores of the different groups evaluated after Ca/04 challenge. N/E = Not evaluated; Dash (–) are used to separate the scores of each individual animal.

A subset of mice (*n* = 4/group) were humanely euthanized at 7 dpc to collect lungs and nasal turbinates (NTs) and evaluate viral loads by TCID50. Compared to the virus titers from tissues obtained from mice in the Ca/04-vehicle group, a trend towards reduced virus load was observed in mice in the Ca/04-UAWJ280 and Ca/04-oseltamivir groups; however, differences were not statistically significant. In contrast, mice in the Ca/04-combination therapy group showed significantly reduced virus titers in both lung and NT samples compared to the Ca/04-vehicle control group. Overall, these studies are consistent with the clinical outcome of the disease in the various treatment groups and support the notion that UAWJ280 has antiviral activity against Ca/04 (H1N1) and synergizes the activity of oseltamivir (Figure 7(A,B)).

Histopathological analysis of lungs at 7, 14 and 21 dpc revealed minimal variations in lesions severity and distribution among treated groups, with overall milder trends observed in the oseltamivir- and combined-treatment groups. Lesions in the Ca/04-Vehicle group at 7dpc (Figure 7(D)) were centred on the airways and consisted of subacute moderate to severe bronchointerstitial pneumonia with necrotizing bronchiolitis characterized by attenuation and necrosis of the respiratory epithelium and accumulation of fibrinonecrotic exudate within the lumen. Moderate numbers of neutrophils, macrophages, lymphocytes and plasma cells were expanding the bronchial and bronchiolar lamina propria, extending to the submucosa, adjacent peribronchiolar interstitium, perivascular spaces and alveolar septa. Of these, approximately 20-30% were collapsed, discontinuous, necrotic and superimposed by the aforementioned mixed inflammatory cellular infiltrate in association with oedema, fibrin and haemorrhage. The pleura was multifocally lined by hyperplastic mesothelium and occasionally infiltrated by small numbers of mixed inflammatory cells. In all the treated groups at 7 dpc, lesions reiterated what was observed in the vehicle-Ca04wt with a variable degree of severity as reported in Figure 6(U). In the Ca/04-UAWJ280 group at 7 dpc (Figure 7(E)), between 40% and 60% of the alveoli were edematous and infiltrated by moderate numbers of macrophages and neutrophils. Small to moderate numbers of lymphocytes and plasma cells were collaring vessels. Necrotizing bronchiolitis with intraluminal fibrinonecrotic exudate accumulation was moderate.

The Ca/04-Oseltamivir and Ca/04-Comb therapy groups at 7 dpc (Figure 7(F,G)) presented a comparable degree and extent of inflammation effacing approximately 30–45% of the total lung section. Lesions were limited at the hilum, well demarcated and rarely extended to the middle of the lung lobe. These were characterized by focally extensive consolidation due to septal collapse and infiltration by moderate numbers of aforementioned inflammatory cell infiltrate. Within these areas, bronchitis and bronchiolitis were moderate with fibrinonecrotic exudate occluding smaller airways. By 14 dpc, all mice from the Ca/04-Vehicle group perished or had to be humanely euthanized. The Ca/04-UAWJ280 group at 14 dpc presented marked epithelization of the lung parenchyma with almost diffuse (up to 80% of the section) pneumocyte type 2 hyperplasia and hypertrophy, alveolar oedema and inflammation, predominantly lymphoplasmacytic, and lesser neutrophilic and histiocytic. Bronchial and bronchiolar epithelium was hyperplastic, irregularly blebbing, sloughing into the lumen (Figure 7(I)). At 14 dpc, lesions in the Ca/04-Oseltamivir and Ca/04-Comb therapy groups were localized to the hilar areas of the lung lobes, well-demarcated and characterized by consolidation and epithelization similar to what described for the Ca/04-UAWJ280**.** The percentage of parenchyma affected, and lesion severity was similar between Ca/04-Oseltamivir and Ca/04-Comb therapy groups and ranged between 30–50% of the entire lung section (Figure 7(J,K)). At 21 dpc, between 40% and 60% of the alveoli in the Ca/04-UAWJ280 group were affected by consolidation, pneumocyte type 2 hyperplasia, and moderate histiocytic and lymphoplasmacytic infiltrations. Lymphocytes and plasma cells were occasionally forming perivascular nodular aggregates organizing into early germinal centres. Bronchial and bronchiolar mucosa appeared mostly normal with rare blebbing and regeneration (Figure 7(M)). Ca/04-Oseltamivir and Ca/04-Comb therapy groups at 21 dpc presented similar extents of lesions than at 14 dpc consistent with moderate consolidation due to parenchyma epithelization and moderate lymphoplasmacytic inflammation. Lymphoid aggregates showing early germinal centre formations were expanding the interstitium adjacent to vessels and airways throughout, especially prominent at the hilar areas. (Figure 7(N,O)).

Variable amounts of influenza virus nucleoprotein were detected by immunostaining in the lungs of all groups except the negative control (Figure 7(P)). Staining was located both in the nucleus and cytoplasm of infected cells. The intensity, number of positive cells and distribution of the virus antigen was overall higher in the Ca/04-Vehicle mice (Figure 7(U)). Staining was mostly centred around airways with the highest positivity within bronchial, bronchiolar epithelial cells, and necrotic cellular exudate forming within the lumen of the airways. Within the Ca/04-Vehicle group, moderate numbers of alveolar macrophages and pneumocytes within the alveolar septa were also positive (Figure 7(Q)). The Ca/04-UAWJ280 mice lungs presented moderate amounts of virus nucleoprotein, predominantly within bronchial epithelial cells and lesser within alveolar macrophages and pneumocytes (Figure 7(R)). The Ca/04-Oseltamivir group presented mild-moderate amounts of viral antigens which were confined to the bronchial epithelium and rare positive cells were observed within the interstitium (Figure 7(S)). The Ca/04-Comb therapy group had the lowest level of positivity among the groups evaluated, with 2 out of 4 mice with minimal amounts of nucleoprotein detected (Figure 7(U)). Viral antigen was confined to the airways epithelium and rarely seen within the alveolar septa and interstitium (Figure 7(T)).

### UAWJ280 is effective against an oseltamivir-resistant variant strain Ca/04 OsR

**Figure 8.**
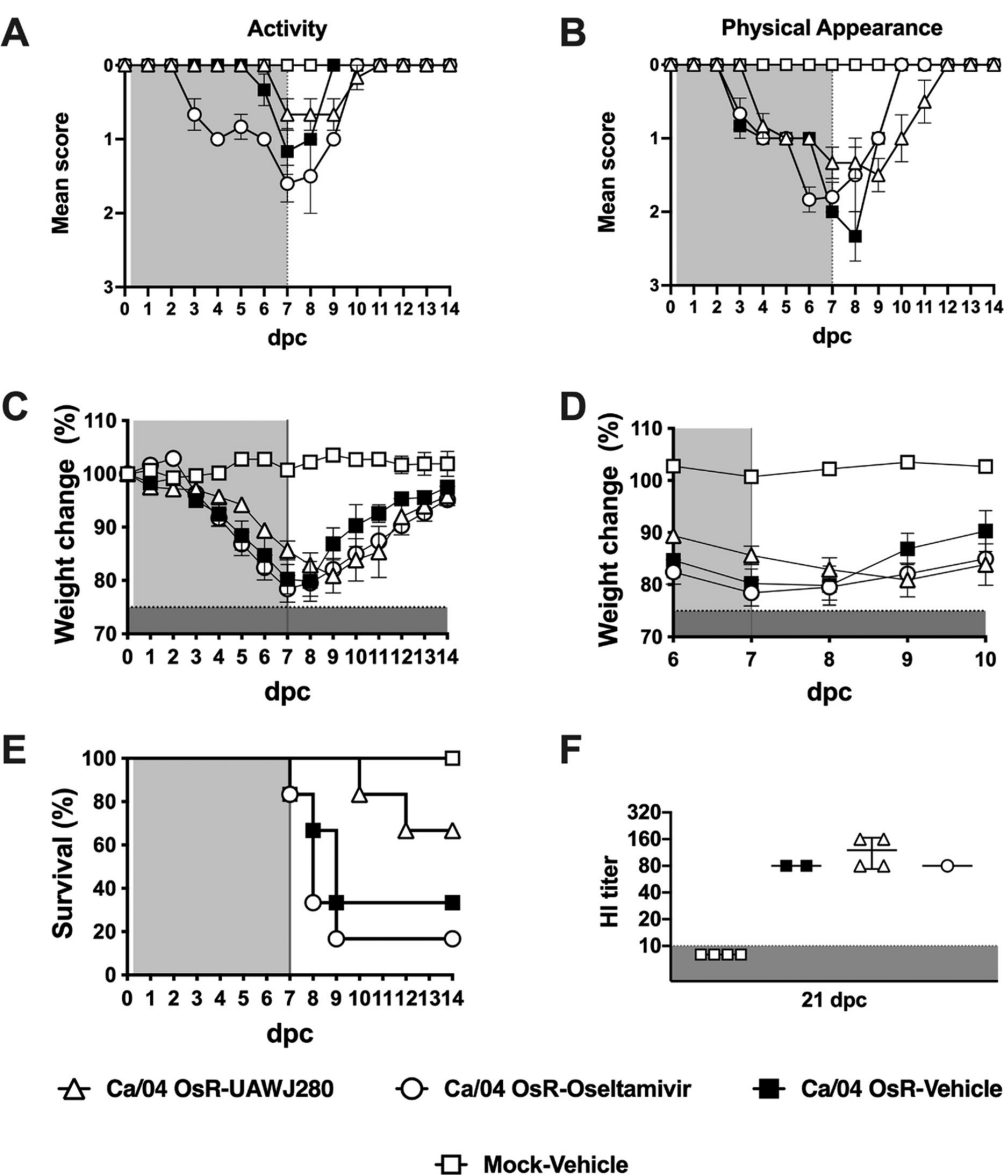
Evaluation of the efficacy of UAWJ280 against an oseltamivir-resistant IAV strain in the mice model. Mice were inoculated with 5xMLD50 of Ca/04 oseltamivir-resistant strain (Ca/04 OsR) and treated with drug vehicle, UAWJ280 (100 mg/kg per dose) and oseltamivir (5 mg/kg per dose) through intraperitoneal injection 4 h post-challenge and continue for 7 days, twice a day (cyan shedding). Clinical signs related to (A) activity, (B) physical appearance, (C-D) weight change and (E) survival were evaluated among the different groups. (F) Sera was recovered at 21 dpc and the levels of neutralizing antibodies in each group was assessed using HI assay.

To further explore the antiviral activity of UAWJ280, we tested its efficacy against a Ca/04 (H1N1) strain carrying the oseltamivir-resistant mutation NA H275Y (Ca/04 OsR). Ca/04 OsR was generated through reverse genetics [37]. Of note, the Ca/04 OsR strain is resistant to both oseltamivir and amantadine since the wild-type Ca/04 is naturally resistant to the latter (Figure 1). Mice (*n* = 6/group) were challenged with 5xMLD50 of Ca/04 OsR and subsequently treated with either UAWJ280 or oseltamivir. As expected, oseltamivir treatment was unable to provide protection of mice challenged with the Ca/04 OsR virus (Figure 8(A–E)). Mice in the Ca/04 OsR-oseltamivir group showed clinical signs, body weight loss, and survival rates similar or worse than mice in the Ca/04 OsR-vehicle group with ∼20% body weight loss in both of these groups and 16% and 33% survival, respectively. In comparison, mice in the Ca/04 OsR- UAWJ280 group showed mild clinical signs, ∼15% body weight loss, and 65% survival. HI assays using sera obtained 21 dpc showed similar levels of neutralizing antibodies against the Ca/04 OsR challenge virus among surviving mice (Figure 8(F)). The results demonstrate that UAWJ280 alleviates the clinical signs associated with the infection of an oseltamivir-resistant H1N1 strain.

## Discussion

Seasonal IAV infections result in about 3–5 million cases of severe disease, and between 300,000 and 500,000 deaths around the world every year [38]. For the US, influenza virus infections result in an average economic impact of $87 billion due to prophylactic, therapeutic and hospitalization costs, and missed school or workdays [39–41]. In addition, the emergence of novel IAV strains of pandemic concern highlights the need for the development of efficacious countermeasures [4]. Common antiviral options against IAVs include adamantanes that target the M2 ion channel, NAIs that bind the NA catalytic site and recently baloxavir, which targets the PA polymerase subunit. Unfortunately, nearly 99% of the IAV strain circulating in humans are resistant to the M2 inhibitors, which render drugs such as amantadine and rimantadine obsolete [12,42]. In addition, it is common to observe the emergence of NAI-resistant strains, particularly in immunocompromised individuals infected with H1N1 strains (NA H275Y mutation) [4,6–8,16,42]. NAI-resistant strains have also emerged from H3N2 and influenza B viruses [43]. Baloxavir marboxil was recently approved by the FDA for use against IAV and IBV infections as a single dose treatment. Baloxavir marboxil targets the cap-dependent endonuclease activity of the PA polymerase subunit of influenza viruses and prevents viral mRNA synthesis [44]. Clinical studies suggest that baloxavir treatment is more effective at reducing the duration of fever and influenza-like symptoms compared to individuals treated with NAIs [45,46]. However, baloxavir marboxil exhibits a low barrier of resistance, with the single PA I38T mutation leading to strains with overall fitness similar to the wild-type strain [47,48].

Here we report the development of a novel antiviral that targets the M2, UAWJ280, that shows efficacy in the presence of the M2-S31N polymorphism. UAWJ280 is a deuterium-containing compound that was designed based on a previously reported M2-S31N inhibitor UAWJ106 [17]. Unexpectedly, UAWJ280 showed improved channel blockage against M2-S31N channel in the TEVC assay and more potent antiviral activity against several IAVs than UAWJ106. *In vivo* PK profiling showed that UAWJ280 had favourable PK properties that warrant further advance to the *in vivo* antiviral efficacy study.

Serial viral passage experiment revealed two M2 mutants, the M2-L26I and M2-L46P, that confer drug resistance against UAWJ280. Interestingly, a previous study similarly discovered L26I and L46P as drug-resistant mutants against the isoxazole M2-S31N inhibitors WJ379 [32] and UAWJ102 (compound 4 in [49]), respectively. The L26I mutant locates at the drug-binding site and might have a direct effect on drug binding (Figure 3(B)). In contrast, the L46P locates far away from the drug-binding site (> 20 Å) and appears to have an allosteric effect on drug binding (Figure 3(B)). It was suggested by the molecular dynamics simulations that the L46P mutant broadened the drug-binding site at the N-terminus of the M2 channel, resulting in reduced binding affinity [49]. Coupled with the results from this study, it appears that L26I and L46P might confer drug resistance against structurally disparate M2-S31N inhibitors, and it is important to test future M2-S31N inhibitors against these two mutants.

Assessment of toxicity related to UAWJ280 alone or in combination with oseltamivir showed no significant toxic effects in mice. Mice treated with either UAWJ280 alone or in combination with oseltamivir remained healthy during and after treatment and behaved not different than mice treated with oseltamivir alone whose safety profile is well documented [50,51]. Histopathological examination of lungs, live, kidney, heart and brain from mice under different treatments showed no signs of toxicity in any of the organs evaluated (Figure 5).

UAWJ280 showed antiviral activity *in vivo* against lethal IAV H1N1 challenge in mice. Influenza challenged mice treated with UAWJ280 showed milder clinical signs and improved survival compared to virus challenge-non-treated control mice. Unlike oseltamivir, UAWJ280 was also effective in mice challenged with the oseltamivir-resistant Ca/04 OsR strain (NA H275Y). Ca/04 OsR-challenged mice treated with UAWJ280 showed reduced clinical signs and increased survival in comparison with similarly challenged controls treated with oseltamivir (or vehicle).

Lungs and NTs were collected from mice in each of the Ca/04-challenge groups at 7 dpc to determine viral loads. Although monotherapy with either UAWJ280 or oseltamivir led to improved clinical outcomes, they only led to a non-statistically significant trend towards reduced virus titers compared to vehicle-treated controls. This observation is not necessarily surprising; it is in fact consistent with previous reports on the use of oseltamivir in mice [52,53]. Studies using the ferret model of influenza suggest that oseltamivir treatment slightly decrease virus load, but such small effect produces a significant effect in reducing virus-induced inflammation, which ultimately produces improved clinical outcomes [54]. More studies are needed to better address similar questions using UAWJ280. Of significance, UAWJ280 enhanced the protective effects of oseltamivir as reflected by significantly improved clinical outcomes and survival of mice compared to those in the monotherapy groups (Figure 6). Treatment with UAWJ280 in combination with oseltamivir led to a significant reduction in virus loads in both lungs and NTs at 7 dpc. Dual therapies with oseltamivir and amantadine have proven more effective in mice challenged with amantadine-sensitive IAV strains [55,56]. Dual therapies with combinations of oseltamivir/favipiravir and oseltamivir/ribavirin were shown more effective in mice than treatment with oseltamivir monotherapy against H1N1 and H5N1 IAV, respectively [52,57]. However, dual therapies do not necessarily prevent the emergence of oseltamivir-resistant variants. Recently, a multicenter, double-blind, randomized phase II clinical trial was published that showed reduced virus shedding at day 3 in patients that were administered a trivalent therapy (oseltamivir/amantadine/ribavirin, 5-day treatment course) compared to those under oseltamivir monotherapy. Interestingly, this difference was not associated with improved clinical benefits for reasons yet to be elucidated [53]. The discrepancy between the benefits in clinical outcomes of combination therapies in mice and double and/or triple therapies in humans are likely inherent to the complexities of influenza infections in the latter [53].

The efficacy of UAWJ280 serves as proof-of-principle that antivirals designed against the M2 carrying the S31N mutation are a valid alternative for the development of antivirals against IAV. Although several reports have shown the antiviral activity of M2-S31N inhibitors against both oseltamivir sensitive and -resistant IAVs [14,15,17–24], to the best of our knowledge, this is the first report providing the *in vivo* antiviral efficacy of M2-S31N inhibitors. Future studies are needed to explore the efficacy of UAWJ280 against different IAV subtypes including highly pathogenic H5 or H7 viruses in different animal models as well as its potential to enhance various other approved anti-influenza antivirals.
